# Comparison of costs associated with transcatheter mitral valve repair: PASCAL vs MitraClip in a real-world setting

**DOI:** 10.1186/s12913-023-09966-8

**Published:** 2023-09-04

**Authors:** Jean Marc Haurand, Jafer Haschemi, Daniel Oehler, Yvonne Heinen, Amin Polzin, Malte Kelm, Patrick Horn

**Affiliations:** 1 Department of Cardiology, Pulmonology and Vascular Medicine, University Hospital Düsseldorf, Medical Faculty of the Heinrich Heine University Düsseldorf, Moorenstr. 5, 40225 Düsseldorf, Germany; 2 CARID, Cardiovascular Research Institute, Medical Faculty and University Hospital Düsseldorf, Heinrich-Heine University, Düsseldorf, Germany

**Keywords:** Mitral valve regurgitation, Cost comparison, MitraClip, PASCAL, Complications

## Abstract

**Aims:**

We aimed to conduct a clinical process cost analysis to evaluate all upcoming costs of mitral valve transcatheter edge-to-edge repair (M-TEER) treatment using the MitraClip and the PASCAL repair system.

**Methods:**

First, we prospectively enrolled 107 M-TEER patients treated with either the PASCAL or MitraClip system and compared all upcoming costs during the M-TEER procedure and the associated in-hospital stay. Second, we retrospectively analysed 716 M-TEER procedures with regard to the occurrence of complications and their associated costs. All materials used in the catheterization laboratory for the procedures were evaluated. The cost analysis considered various expenses, such as general in-hospital costs, device costs, catheter laboratory and material costs.

**Results:**

In the prospective study, 51 patients were treated using the PASCAL system, and 56 were treated using the MitraClip system. The two groups had comparable baseline characteristics and comorbidities. The total in-hospital costs were 25 414 (Interquartile range (IQR) 24 631, 27 697) € in the PASCAL group and 25 633 (IQR 24 752, 28 256) € in the MitraClip group (*p* = 0.515). The major cost driver was initial material expenditure, mostly triggered by device costs, which were similar to the PASCAL and MitraClip systems. Overall intensive care unit and general ward costs did not differ between the PASCAL and MitraClip groups. In the retrospective analysis, M-TEER-related complications were rare but were associated with higher costs, mainly due to prolonged hospitalisation.

**Conclusion:**

The major cost driver of M-TEER was the material expenditure, which was mostly triggered by high device costs. The costs of treating patients were similar for the PASCAL and MitraClip systems**.** M-TEER-related complications are associated with higher costs, mainly due to prolonged hospitalisation. This analysis provides valuable insights into reducing expenses by modifying the process of M-TEER.

**Supplementary Information:**

The online version contains supplementary material available at 10.1186/s12913-023-09966-8.

## Introduction

Transcatheter edge-to-edge repair (M-TEER) of the mitral valve has emerged as a standard treatment for select patients with clinically relevant mitral regurgitation (MR) and increased surgical risk [1, 2]. Despite the lack of data on the actual expenses of M-TEER treatment so far, it is crucial to conduct a thorough investigation into the real costs associated with the procedure, including device costs, hospitalization costs, and any additional expenses due to complications. Such an analysis would provide valuable insights that enable to identify opportunities for cost reduction while maintaining or even improving the quality of patient care; Through the identification of inefficient processes, healthcare providers can optimize workflows, remove redundancies, and streamline M-TEER treatment, leading to significant cost savings. Additionally, by recognizing cost drivers and comparing different M-TEER systems, they can make well-informed decisions regarding the selection of available options. Cost analyses further offer insights into variations in clinical practices that could affect overall costs.

Therefore, we conducted clinical process cost analysis to evaluate prospectively the total costs of M-TEER treatment using the current two most widely used M-TEER systems, the MitraClip (Abbott Vascular, Santa Clara, CA, USA) and the PASCAL repair system (Edwards Lifesciences Corp., Irvine, CA, USA), within the German healthcare system. Given the low rate of M-TEER related complications, we conducted an additional retrospective analysis of M-TEER procedures to investigate the occurrence of complications and their associated costs.

## Methods

First, we prospectively compared the two available M-TEER systems, the MitraClip and the PASCAL repair system, regarding all upcoming costs during the M-TEER procedure and the associated in-hospital stay. This prospective study enrolled 107 M-TEER patients treated with the PASCAL or MitraClip system at our university hospital between 2020 and 2021. After examining patients as a group in the presence of both surgeons and interventional cardiologists, the heart team discussed all cases. To evaluate the individual risk of surgery and inform therapeutic decision-making, the team used risk calculators such as EuroScore II and Society of Thoracic Surgeons (STS) risk score, as well as frailty assessments and Charlson comorbidity score. Frailty status was evaluated using the Fried criteria and based on five components: unintentional weight loss, weakness, exhaustion, slowness, and low physical activity. For each criterion met to specification, 1 point was scored. Patients who met at least 3 of the 5 criteria were classified as frail. The patient selection criteria for M-TEER mandated that patients exhibit either symptomatic severe MR at rest or moderate MR at rest that progressed to a severe degree during exercise, adhering to the relevant thresholds for severe MR as per the guidelines valid during the study [3]. Functional MR patients were expected to meet the ventricular parameter requirements based on the COAPT criteria [4]. Each patient was assigned to the next available implantation date with alternating weekly time slots for PASCAL and MitraClip. The treating physicians did not influence scheduling or system selection.

Secondly, we conducted a retrospective analysis of 709 M-TEER patients treated between the years 2010 and 2020. The analysis focused on M-TEER-related complications and their associated costs, including pericardial tamponade, stroke, acute kidney injury (AKI), and vascular complications.

The implantation technique for M-TEER has been described in detail previously [4]. All M-TEER procedures were performed without using ultrasound-guided venous puncture or vascular closure devices.

All materials used in the catheterization laboratory for the procedures, including sterile drapes, catheters, sheaths, guidewires, closure devices, and other sterile and non-sterile materials, were evaluated. The cost analysis considered various expenses, such as general in-hospital costs, technology costs, catheter laboratory and material costs (covering equipment and anaesthesia costs), intensive care unit (ICU) stay costs, general ward costs, and additional expenses (such as blood products and complications). For device costs, the non-discounted recommended retail price of the device was used. The material and cost variables were utilized to determine the primary endpoint of M-TEER by computing the total costs. The safety endpoints of this study (including bleeding, stroke and AKI) were defined according to the Mitral Valve Academic Research Consortium [5]. AKI was defined as an increase in serum creatinine of ≥ 0.3 mg/dl within 48 h compared with baseline or an increase in serum creatinine to ≥ 1.5 times baseline within seven days.

Results are expressed as mean ± standard deviation (SD) or median with interquartile range (IQR). The Shapiro–Wilk test was used to assess the normal distribution of the parameters. Patient characteristics were compared using an unpaired *t*-test (continuous normally distributed data), two-tailed Fisher’s exact test (categorical data with two variables), and Pearson’s chi-squared test (categorical data with more than two variables). The Mann–Whitney U test was used to compare non-normally distributed data. Statistical significance was set at *P* < 0.05. Statistical analyses were performed using SPSS Statistics 28 (IBM®) and Prism (GraphPad®).

## Results

### Prospective

#### Patient characteristics

In total, 51 patients were treated using the PASCAL system, and 56 were treated using the MitraClip system. The two groups had comparable baseline characteristics and comorbidities (Table 1). The median patient age was 81 (IQR 76, 83) years in the PASCAL group and 79 (IQR 73, 82) years in the MitraClip group (*p* = 0.206). The EuroSCORE II was similarly high in both groups: 5.0 (IQR 3.7, 8.5) in the PASCAL group vs 6.3 (IQR 4.3, 8.3) in the MitraClip group (*p* = 0.306). In the PASCAL group, 24 out of 51 patients (47%) were classified as frail, while in the MitraClip group, 25 out of 56 patients (45%) were classified as frail (*p* = 0.802). The Charlson Comorbidity Score showed no difference between the groups, with values of 5.0 (4.0, 6.0) in the PASCAL group and 5.0 (4.0, 7.0) in the MitraClip group (*p* = 0.101).

**Table 1:** Patient characteristics of MR patients grouped according to the device system used for M-TEER. Values are n (%) or median (interquartile range). * indicates *p* ≤ 0.05 between the groups

	**PASCAL** **(*n* = 51)**	**MitraClip** **(*n* = 56)**	***p*-value**
**Baseline characteristics**			
Age (years)	81 (76, 83)	79 (73, 82)	0.206
Female, n (%)	26 (51.0)	23 (41.1)	0.304
EuroSCORE II (%)	5.0 (3.7, 8.5)	6.3 (4.3, 8.3)	0.306
STS risk score (%)	5.3 (3.9, 8.1)	5.1 (3.1, 7.7)	0.244
Frailty, n (%)	24 (47.1)	25 (44.6)	0.802
CCI (points)	5.0 (4.0, 6.0)	5.0 (4.0, 7.0)	0.101
NYHA functional class, n (%)			0.445
II	8 (15.7)	12 (21.4)	
III	40 (78.4)	38 (67.9)	
IV	3 (5.9)	6 (10.7)	
Comorbidities, n (%)			
- Arterial hypertension	45 (88.2)	44 (78.6)	0.182
- Diabetes mellitus	14 (27.5)	13 (23.3)	0.614
- Coronary artery disease	35 (68.6)	37 (66.1)	0.778
- Previous myocardial infarction	7 (13.7)	7 (12.5)	0.851
- Previous cardiac surgery	15 (29.4)	22 (39.3)	0.283
- ICD/ CRT	11 (21.6)	9 (16.1)	0.466
- Atrial fibrillation	38 (74.5)	47 (83.9)	0.229
- Chronic lung disease	8 (15.7)	11 (19.6)	0.593
- Peripheral artery disease	10 (19.6)	9 (16.7)	0.633
- Dialysis for end-stage renal disease	1 (2.0)	1 (1.8)	0.947
- Prior Stroke, n (%)	3 (5.9)	4 (7.1)	0.792
Estimated GFR (ml/min)	51 (35, 65)	50 (40, 62)	0.835
NT-proBNP (*1000 pg/ml)	2.40 (1.05, 3.99)	1.84 (0.99, 5.42)	0.865
Haemoglobin (g/dl)	12.9 (11.2, 13.7)	12.6 (11.4, 13.7)	0.865

*MR* Mitral regurgitation, *M-TEER* Transcatheter mitral valve edge-to-edge repair, *STS* Society of Thoracic Surgeons, *CCI* Charlson Comorbidity Index, *NYHA* New York Heart Association, *ICD* Internal cardiac defibrillator, *CRT* Cardiac resynchronization therapy, *GFR* Glomerular filtration rate, *NT-proBNP* Brain natriuretic peptide

At baseline, 84.3% of the patients in the PASCAL group and 78.6% of the patients in the MitraClip group suffered from New York Heart Association (NYHA) functional Class III or IV (*p* = 0.234). Additionally, the groups did not differ in terms of echocardiography-derived ventricular or valvular parameters (Table 2). All patients had dynamic moderate-to-severe or severe MR at baseline. Functional MR was more common than degenerative MR in both groups: 72.6% of patients in the PASCAL group and 69.6% of patients in the MitraClip group had functional MR (*p* = 0.945). Patients in both groups also had similar left ventricular ejection fraction (LVEF) measurements: In the PASCAL group, 39.2% of patients had LVEF < 40% and 39.2% of patients had LVEF > 50%. In the MitraClip group, 35.8% of patients had LVEF < 40% and 44.6% of patients had LVEF > 50% (*p* = 0.681). Finally, left ventricular diastolic diameter did not differ between the groups (56 [IQR 46, 65] mm in the PASCAL group and 55 [IQR 52, 65] mm in the MitraClip group [*p* = 0.541]).

**Table 2:** Baseline echocardiographic and hemodynamic parameters of MR patients grouped according to the device system used for M-TEER. Values are n (%) or median (interquartile range). * indicates *p* ≤ 0.05 between the groups

	**PASCAL** (*n* = 51)	**MitraClip** (*n* = 56)	***p*-value**
MR etiology, n (%)			0.945
Functional MR	37 (72.6)	39 (69.6)	
Degenerative MR	10 (19.6)	12 (21.4)	
Mixed dissease	4 (7.8)	5 (9.0)	
MR severity, n (%)			0.906
Moderate (at rest)	3 (5.9)	3 (5.4)	
Severe	48 (94.1)	53 (94.6)	
Left ventricle			
Median LVEF (%)	47 (33, 55)	52 (30, 59)	0.681
- LVEF < 40%, n (%)	20 (39.2)	20 35.8)	
- LVEF 40–50%, n (%)	12 (21.6)	11 19.6)	
- LVEF > 50%, n (%)	20 (39.2)	25 (44.6)	
LVEDD (mm)	56 (46, 65)	55 (52, 65)	0.541
Left atrium area (cm2)	27 (21, 34)	27 (22, 32)	0.821
Transmitral gradient (mmHg)	2 (1, 3)	2 (1, 3)	0.419
Vena contracta (mm)	7 (5, 8)	7 (6, 8)	0.229
EROA (cm^2^)	0.31 (0.25, 0.44)	0.30 (0.25, 0.40)	0.212
Regurgitation volume (ml)	47 (40, 62)	52 (42, 64)	0.105
Right Ventricle			
RVEDD (mm)	32 (26, 38)	32 (28, 41)	0.241
TAPSE (mm)	18 (15, 21)	17 (13, 20)	0.094
Hemodynamic parameters			
Cardiac Index	2.0 (1.8, 2.3)	2.2 (1.8, 2.6)	0.196
sPAP (mmHg)	46 (34, 63)	49 (34, 57)	0.950

*M-TEER* Transcatheter mitral valve edge-to-edge repair, *MR* Mitral regurgitation, *LVEF* Left ventricular ejection fraction, *LVEDD* Left ventricular end-diastolic diameter, *RVEDD* Right ventricular end-diastolic diameter, *EROA* Effective regurgitation orifice area, *TAPSE* Tricuspid annular plane systolic excursion, *sPAP* systolic pulmonary artery pressure

#### Procedural outcome

Technical success was achieved in 98% of patients in the PASCAL group and 100% in the MitraClip group (*p* = 0.474). One patient in the PASCAL group had no implanted device because of an inadequate mitral valve orifice area. There was no significant difference in the number of implanted devices (1 [IQR 1, 1] vs. 1 [IQR 1, 2], *p* = 0.218) or mean valvular gradient (4 [IQR 3, 4] mmHg vs. 4 [IQR 2, 5] mmHg, p = 0.833). Procedural durations were similar between the groups (95 [IQR 70, 120] min vs 90 [IQR 60, 121] min, *p* = 0.389).

M-TEER safety was similar in both groups, with no cardiac structural damage, stroke, or conversion to open-heart surgery (Table 3). The rate of AKI was similar in both groups: 4 out of 51 (7.8%) patients suffered from AKI in the PASCAL group, as well as 4 out of 56 patients in the MitraClip group (*p* = 0.891). Minor bleeding occurred in two patients (3.9%) in the PASCAL group and one patient (1.8%) in the MitraClip group (*p* = 0.608). Major bleeding occurred in one patient (1.8%) in the MitraClip group. Bleeding complications mainly occurred at access sites closed by a z-shaped suture and were successfully managed by manual compression. Three patients in the PASCAL group (5.9%) and two patients (3.6%) in the MitraClip group experienced acute kidney injury post-procedural, which resulted in prolonged hospital stays. None of the patients in either group died during the procedures or their hospital stays.

**Table 3:** Procedural outcome. Values are n (%) or median (interquartile range). * indicates *p* ≤ 0.05 between the groups

	**PASCAL** (*n* = 51)	**MitraClip** (*n* = 56)	***p*-value**
Procedure duration (min)	95 (67, 120)	90 (60, 121)	0.389
Conversion to surgery, n (%)	0 (0)	0 (0)	
Periprocedural mortality, n (%)	0 (0)	0 (0)	
Pericardiocentesis, n (%)	0 (0)	0 (0)	
Leaflet device detachment, n (%)	0 (0)	0 (0)	
Technical success, n (%)	50 (98)	56 (100%)	0.474
Minor bleeding complication, n (%)	2 (3.9)	1 (1.8)	0.608
Major vascular complication, n (%)	0 (0)	1 (1.8)	
Myocardial Infarction, n (%)	0 (0)	0 (0)	
Pneumonia, n (%)	2 (3.9)	2 (3.6)	0.924
Acute kidney failure, n (%)	4 (7.8)	4 (7.1)	0.891
Stroke 30 days, n (%)	0 (0)	0 (0)	
Intrahospital mortality, n (%)	0 (0)	0 (0)	
Devices implanted, n (%)			0.157
0	1 (2)	0 (0)	
1	38 (74.5)	36 (64.3)	
2	12 (23.5)	19 (33.9)	
3	0 (0)	1 (1.8)	
Degree of MR at discharge, n (%)			0.951
mild	35 (68.6)	40 (71.4)	
moderate	15 (29.4)	15 (26.8)	
severe	1 (2.0)	1 (1.8)	
Transmitral gradient at discharge (mmHg)	4 (3, 4)	4 (2, 5)	0.833
Length of stay in the ICU (d)	1 (1, 2)	1 (1, 1)	0.164
Total length of hospital stay (d)	5 (4, 10)	6.5 (4, 10)	0.239

*MR* Mitral regurgitation, *ICU* Intensive care unit

The length of ICU stay did not differ between the groups (PASCAL group:1 [IQR 1, 2] day vs MitraClip group:1 [IQR 1, 1] day, *p* = 0.164). Furthermore, the total length of hospital stay was similar between groups (5 [IQR 4, 10] days vs 6.5 [IQR 4, 10] days, *p* = 0.239).

Over the one-year follow-up period, hospitalization for heart failure occurred in 7 patients (14%) after PASCAL implantation and in 10 patients (18%) after MitraClip implantation (*p* = 0.559).

#### Cost analysis of PASCAL vs MitraClip

**Fig. 1:**
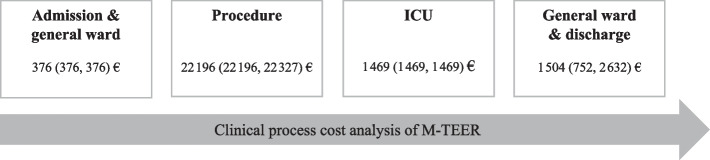
Clinical process cost analysis of transcatheter mitral valve repair (M-TEER)**.** The figure shows the clinical process of M-TEER and associated costs of the pre-procedural costs of the general ward, the costs of the M-TEER procedure (device costs, staff expenses of the catheter laboratory team, and additional material), and the post-procedural costs of the post-procedural care on intensive care unit (ICU) and general ward in the full cohort

**Fig. 2:**
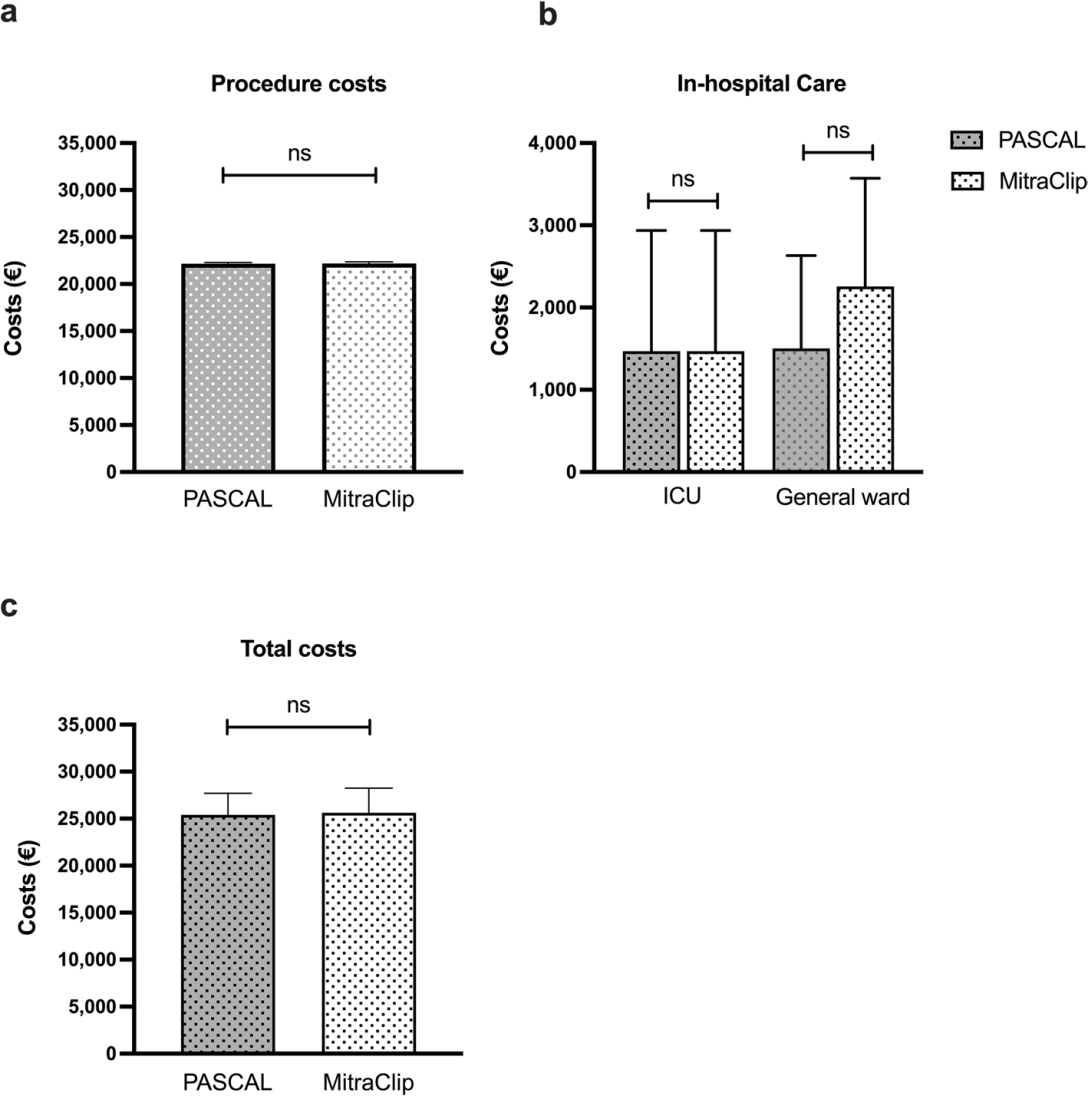
Comparison of costs associated with transcatheter mitral valve repair (M-TEER) with Pascal vs MitraClip systems. The figure shows **a** the costs of the M-TEER procedure (device costs, staff expenses of the catheter laboratory team, and additional material), **b** the costs of in-hospital care in the intensive care unit (ICU) and general ward care, and that **c** the total costs of M-TEER treatment did not differ between patients treated with PASCAL or MitraClip

The clinical process cost analysis for the full cohort included the following components: 376 (376, 376) € for admission and pre-procedural general ward care; 22 196 (IQR 22 196, 22 317) € for procedure costs, which encompassed device costs, staff expenses of the catheter laboratory team, and additional material costs; 1 469 (IQR 1 469, 1 469) € for post-procedural ICU care; and 1 504 (IQR 752, 2 632) € for general ward costs until discharge (Fig. 1). The major cost driver was initial material expenditure, mostly triggered by high device costs which were similar to the PASCAL and MitraClip systems. (Table 4). Both devices had comparable staff expenses for the catheter laboratory team as well as expenses for accessories and supplies, resulting in similar total procedure costs. In the PASCAL group, the total procedure cost was 22 173 (IQR 22 100, 22 304) € while in the MitraClip group, it was 22 200 (IQR 22 100, 22 304) € (*p* = 0.565) (Fig. 2a, Table 4). Overall ICU costs did not differ between the PASCAL and MitraClip groups (1 469 [IQR 1 469, 2 938] € vs. 1 469 [IQR 1 469, 1 469] €, *p* = 0.109). There was a trend toward lower general ward costs in the PASCAL group compared to the MitraClip group (1 504 [IQR 1 128, 2 632] € vs 2 068 [IQR 1 128, 3 572] €, *p* = 0.052) (Fig. 2b, Table 4). The costs associated with transfusion of packed red blood cell units did not differ between groups (170 vs 255 €, *p* = 1). The total in-hospital costs were 25 414 (IQR 24 631, 27 697) € in the PASCAL group and 25 633 (IQR 24 752, 28 256) € in the MitraClip group (*p* = 0.515) (Fig. 2c, Table 4).

**Table 4:** General costs of M-TEER treatment. Values are median (interquartile range). * indicates *p* ≤ 0.05 between the groups

	**Full cohort** (*n* = 107)	**PASCAL** (*n* = 51)	**MitraClip** (*n* = 56)	***p*-value**
Total costs procedure (€)	22,196 (22,101, 22,327)	22,173 (22,100, 22,304)	22,200 (22,100, 22,304)	0.565
Staff costs (€)	484 (417, 632)	495 (423, 627)	480 (409, 647)	0.406
Device costs (€)	21,000	21,000	21,000	1.000
Costs ICU (€)	1468 (1469, 1469)	1469 (1469, 2938)	1469 (1469, 1469)	0.109
Costs general ward (€)	1880 (1128, 3008)	1504 (1128, 2632)	2256 (1128, 3572)	0.052
Total overall costs (€)	25,500 (24,693, 28,085)	25,414 (24,631, 27,697)	25,633 (24,752, 28,256)	0.515

*M-TEER* Transcatheter mitral valve edge-to-edge repair, *ICU* Intensive care unit

When comparing patients with degenerative MR to those with functional MR, we found that the procedural outcome and costs of M-TEER treatment did not exhibit any significant differences between the two groups (Supplemental Tables 1–4).

#### Retrospective cost analysis of complications

M-TEER is a safe procedure with low complication rates. Therefore, we retrospectively analysed 716 patients who underwent M-TEER (172 patients with the PASCAL system, and 544 patients with the MitraClip system) at our centre to gain further insight into the associated additional cost factors. Patients baseline characteristics were shown in Table 5, echocardiographic parameters and procedural outcome in Supplemental Tables 5 and 6).

**Table 5:** Patient characteristics of M-TEER patients in retrospective all comer cohort. Values are n (%) or median (interquartile range)

	**Total cohort (*n* = 716)**
**Baseline characteristics**	
Age (years)	78 (72, 83)
Female, n (%)	319 (44.6)
EuroSCORE II (%)	4.9 (3.6, 8.2)
NYHA functional class, n (%)	
I	19 (2.7)
II	105 (14.7)
III	490 (68.4)
IV	102 (14.3)
Comorbidities, n (%)	
- Arterial hypertension	626 (87.4)
- Diabetes mellitus	207 (28.9)
- Coronary artery disease	472 (65.9)
- Previous myocardial infarction	142 (19.8)
- Previous cardiac surgery	49 (6.8)
- ICD/ CRT	169 (23.6)
- Atrial fibrillation	462 (64.5)
- Chronic lung disease	143 (20)
- Peripheral artery disease	88 (12.3)
- Dialysis for end-stage renal disease	15 (2.1)
Estimated GFR (ml/min)	49 (35, 64)
NT-proBNP (*1000 pg/ml)	2.48 (1.36, 5.14)
Haemoglobin (g/dl)	12.1 (10.7, 13.4)

*M-TEER* Transcatheter mitral valve edge-to-edge repair, *NYHA* New York Heart Association, *ICD* Internal cardiac defibrillator, *CRT* Cardiac resynchronization therapy, *GFR Glomerular filtration rate, NT-proBNP* Brain natriuretic peptide

**Table 6:** Retrospective analysis of costs associated with complications after M-TEER. Values are median (interquartile range). * indicates *p* ≤ 0.05 between the groups

	** + AKI** (*n* = 70)	**- AKI** (*n* = 646)		***p*-value**
Length of stay in the ICU (d)	1 (1, 2)	1 (1, 2)		0.446
Total length of hospital stay (d)	9 (7, 15)	7 (5, 12)		0.030*
Costs ICU (€)	1469 (1469, 2938)	1469 (1469, 2938)		0.446
Costs general ward (€)	3008 (2162, 5264)	2256 (1128, 3760)		0.013*
	** + Stroke** (*n* = 5)	**- Stroke** (*n* = 711)		
Length of stay in the ICU (d)	1 (1, 4)	1 (1, 2)		0.600
Total length of hospital stay (d)	11 (3, 40)	7 (5, 12)		0.014*
Costs ICU (€)	1469 (1469, 5876)	1469 (1469, 2938)		0.600
Costs general ward (€)	6392 (752, 13,348)	2256 (1128, 3760)		0.016*
Costs neurologic imaging (€)	1390 (695, 1540)	0		
	** + Pericardial tamponade **(*n* = 4)	**- Pericardial tamponade **(*n* = 712)		
Length of stay in the ICU (d)	4 (2, 7)	1 (1, 2)		0.017*
Total length of hospital stay (d)	11 (5, 18)	7 (5, 12)		0.792
Costs ICU (€)	5142 (3305, 10,283)	1469 (1469, 2938)		0.017*
Costs general ward (€)	3196 (564, 4136)	2258 (1128, 3760)		0.712
Costs pericardiocentesis (€)	836 (645, 908)	0		
	**Major bleeding **(*n* = 7)	**Minor bleeding **(*n* = 68)	-** bleeding **(*n* = 640)	
Length of stay in the ICU (d)	1 (1, 3)	1 (1, 2)	1 (1, 2)	0.622
Total length of hospital stay (d)	12 (10, 32)	11 (11, 16)	7 (5, 11)	0.001*
Costs ICU (€)	1469 (1469, 4407)	1469 (1469, 2938)	1469 (1469, 2938)	0.622
Costs general ward (€)	4136 (3384, 11,280)	3760 (2256, 5546)	2256 (1128, 3760)	0.001*
Costs bleeding management (€)	910 (825, 1080)	170 (106, 170)	0	

*M-TEER* Transcatheter mitral valve edge-to-edge repair, *ICU* Intensive care unit

AKI occurred in 70 out of 716 (10.8%) patients after M-TEER (Table 6). The incidence AKI was 8.7% in patients treated with PASCAL and 10.1% in patients treated with MitraClip (*p* = 0.593).

AKI was associated with prolonged hospitalisation: Patients with AKI had more extended hospital stays than those without AKI (9 [IQR 7, 15] days vs 7 [IQR 5, 12] days, *p* = 0.030). The costs of general ward accommodation were higher in patients with AKI (3 008 [IQR 2 162, 5 264] € vs 2 256 [IQR 1 128, 3 760] €, *p* = 0.013). Haemodialysis was required in 2 out of 70 patients with AKI, which was associated with additional costs of 520 € for both cases.

Stroke occurred in 5 out of 716 (0.7%) patients after M-TEER (Table 6) (in 0.6% in patients treated with PASCAL and in 0.7% in patients treated with MitraClip [*p* = 0.833]). The occurrence of stroke was associated with prolonged hospitalisation and higher costs of general ward accommodation [patients with stroke: 11 (IQR 3, 40) days, 6 392 (IQR 752, 13 348) € vs patients without stroke: 7 (IQR 5, 12) days, respectively, 2 256 (IQR 1 128, 3 760) €, (*p* = 0.014, 0.016). In addition, in patients with stroke, cerebral imaging was associated with additional costs of 1 390 (IQR 695, 1 540) € (Table 6).

Pericardial tamponade occurred in 4 out of 716 (0.6%) patients (Table 6) (in 0.6% in patients treated with PASCAL and 0.6% in patients treated with MitraClip [p = 0.963]). In three patients, pericardiocentesis was performed during the procedure and in one patient after the procedure. In all of the four cases pericardiocentesis was sufficient to stabilize the patient until the bleeding stopped spontaneously. Pericardiocentesis was associated with an additional cost (material and staff expenses) of 836 (645, 908) €. In addition, the length of ICU stay was longer in patients with pericardial tamponade than in those without pericardial tamponade: 4 (IQR 2, 7) days vs 1 (IQR 1, 2) (p = 0.017). This was associated with higher ICU stay costs (5 142 (IQR 3 305, 10 283) € vs 1 469 (IQR 1 469, 2 938) €, *p* = 0.017) (Table 6).

Major access site bleeding occurred in seven (1.0%) patients and minor bleeding in 68 out of 716 (9.5%) patients (Table 6). Major bleeding occurred in 1.2% in patients treated with PASCAL, and in 0.9% of patients treated with MitraClip (p = 0.777). The incidence of minor bleeding was 9.9% in patients treated with PASCAL and 9.4% in patients treated with MitraClip (*p* = 0.843). Bleeding was associated with prolonged hospitalisation: The length of hospital stay was 12 (IQR 10, 32) days in patients with major bleeding, 11 (IQR 7, 16) days in patients with minor bleeding, and 7 (IQR 5, 11) days in patients without bleeding complications (*p* = 0.001). Bleeding complications were associated with higher costs of general ward accommodation: 4,136 (IQR 3384, 11,280) € in patients with major bleeding, 3 760 (IQR 2 256, 5 546) € in patients with minor bleeding, and 2 256 (IQR 1 128, 3 760) € in patients without bleeding complications, *p* = 0.001). Additional imaging and management of bleeding complications (e.g. transfusion of packed red blood cells) added costs of 910 (IQR 825, 1 080) € in patients with major bleeding and 170 (IQR 106, 170) € in patients with minor bleeding (Table 6).

## Discussion

To our knowledge, this is the first report of a clinical process cost analysis that evaluates the real costs associated with the procedure, including device costs, hospitalization costs, and any additional expenses due to complications. We demonstrated that 1) the major cost driver was initial material expenditure, triggered mainly by device costs; 2) the costs of treating patients with M-TEER were similar between the PASCAL and MitraClip systems; and 3) M-TEER-related complications were rare but associated with higher costs mainly due to prolonged hospitalisation.

### Cost-effectiveness of M-TEER

MR is one of the most common valvular heart diseases worldwide. Its prevalence increases with age and exceeds the prevalence of aortic valve disease [6, 7]. M-TEER has emerged as a standard treatment in selected patients with clinically relevant MR and increased surgical risk related to the type of MR [1, 2]. M-TEER is an expensive procedure mainly driven by high device costs, as demonstrated in the present study, which is an issue for every healthcare system. However, data and sub-analysis from the COAPT trial showed that despite guideline-directed medical therapy (GDMT), M-TEER using MitraClip for patients with functional MR and heart failure leads to an overall gain of additional life-years, as well as quality-adjusted life years, compared to GDMT alone [4, 8]. In addition, compared with medical therapy alone, M-TEER resulted in a lower rate of hospitalisation due to heart failure within 24 months of follow-up [4]. We previously demonstrated that the beneficial long-term effects of M-TEER in nonagenarians were comparable to those in younger patients [9]. These clinical benefits for patients might reduce the costs of healthcare systems. It has been demonstrated that M-TEER can be cost-saving for healthcare systems in countries such as the United States, the United Kingdom, and Germany [8, 10, 11]. In a systematic review analysing the cost-effectiveness of M-TEER, it improved both life expectancy and quality-adjusted life years compared to GDMT and led to an increased cost-effectiveness ratio with willingness-to-pay thresholds higher than the cost per quality-adjusted life years gained by treatment in selected countries [12]. In a prospective single-armed registry with a follow-up of two years, Willits et al. showed that M-TEER is associated with reduced hospital readmissions, leading to cost savings for the healthcare system in the United Kingdom [13]. Taken together, M-TEER is expensive but might be cost-saving, depending on the conditions of single patients treated with M-TEER and the health care system.

### M-TEER costs in detail using PASCAL vs MitraClip

In addition to the above mentioned cost-effectiveness studies, we provide a more detailed breakdown of M-TEER-associated costs by prospectively assessing associated resources and costs related to a M-TEER hospital stay. Furthermore, we demonstrated that the costs of the different M-TEER systems (PASCAL vs MitraClip) did not differ significantly between systems. The main cost driver was the price of the M-TEER system, which was the same for both device systems in this centre. The device costs depend on individual contracts between the manufacturers and centres. The bundle delivered with each technology kit was comparable in both groups, with comparable accessory materials included. One difference between the systems is the separately provided reusable stabiliser of the MitraClip system, leading to an insignificantly greater expense for sterilisation. The procedural durations and the trained staff needed for M-TEER were comparable for both devices resulting in no differences in total procedure costs. These findings align with previous studies that have compared the two devices, which also reported similar procedural times [14, 15]. Procedural characteristics are relatively minor contributors to the total expenses when compared to the substantial costs incurred by the device kit and critical care accommodations.

Our new data can assist coordinators of M-TEER programs in evaluating and reducing expenses by modifying the processes; Identifying the device kit as the primary cost driver enables effective negotiations aimed at reducing the kit price, securing discounts, and encouraging the companies to develop less-expensive systems, resulting in significant reductions in total M-TEER costs. In addition, the duration of in-hospital care, especially in the ICU, was one of the main cost drivers, with the highest costs per day observed in our study. For both devices, the total length of in-hospital stay was comparable. One day of ICU observation was mandatory in our centre for patients undergoing M-TEER under deep sedation. With growing experience and in light of limited ICU capacities, further findings evaluating the post-procedural complications and safety might lead to shorter post-procedural observation time in recovery rooms followed by transfer to a general ward with no need for ICU admission. This could generate resources for critically ill patients and save expenses for the healthcare system. With the knowledge gleaned of our analysis, healthcare providers are encouraged to assess the safety implications of omitting or limiting costly steps in the process chain, such as post-care ICU, in future studies.

### Complication-associated costs

The cost of M-TEER treatment may vary for different patients. In our study, approximately 45–47% of the patients are classified as frail. A previous study has shown that frailty is linked with a 32% average rise in hospital expenses for patients undergoing M-TEER [16]. This increase is mainly due to a longer recovery time and higher susceptibility to complications. In a comprehensive M-TEER registry, it was observed that patients who experienced complications tended to be older and in a more critically ill condition compared to those who did not encounter any complications following M-TEER [17].

In the present study, we retrospectively analysed 716 patients who underwent M-TEER at our centre to gain further insight into the associated additional cost factors. We found no significant difference in the rate of complications between the PASCAL and MitraClip systems, which is consistent with previous prospective and retrospective studies comparing these devices [14, 15, 18]. Several studies that utilized the same definitions of complications have reported even higher rates of AKI or vascular complications [19–22]. Another registry has reported lower rates of AKI and bleeding events. The observed disparities can also be attributed to variations in the patient cohort, as well as the potential utilization of ultrasound-guided venous puncture or vascular closure devices, which have the capacity to reduce access-site related bleeding events [23].

While M-TEER is generally a safe procedure with minimal complications, the occurrence of a complication can negatively impact patient outcome. Bleeding events after M-TEER have been shown to adversely affect patient outcomes [21, 22]. Additionally, patients experiencing post-M-TEER bleeding were found to have a higher incidence of acute kidney injury compared to those without bleeding [21]. The presence of acute kidney injury after M-TEER was associated with increased short-term and long-term mortality rates [19, 24].

Beyond the clinical impact on patients, our analysis highlights that complications can lead to a heightened demand for resources and increased costs. Our analysis identified prolonged ICU stays and hospitalizations as the primary cost drivers for complications. We here demonstrate that the prevention and standardised management of complications have high economic value. Our findings can be utilized to reduce costs. For instance, at the time of the retrospective analysis, the venous access-site was closed at the end of the procedure using a z-suture technique; however, vascular closure devices were not employed. Our analysis demonstrates that even minor bleeding increases the costs of M-TEER treatment. This understanding encourages investment in additional measures, such as vascular closure devices, to secure the access site and lower bleeding rates, offering a cost-saving strategy in the long run.

In addition, our analysis might encourage future studies focusing on AKI prevention strategies or identifying optimal postprocedural antithrombotic management after M-TEER to reduce thromboembolic events. The effectiveness of cerebral protection devices in reducing the occurrence of cerebrovascular events during M-TEER procedures remains uncertain, necessitating further investigation through additional studies. These studies not only hold considerable clinical relevance but also have the potential to preserve resources and decrease expenses associated with M-TEER treatment.

### Limitations

This study is a single-centre analysis of the German healthcare system. Thus, cost analysis may vary among centres and healthcare systems with different peri- and post-procedural management and costs. However, these differences may also affect the total cost of the M-TEER procedure independent of the devices used. Furthermore, there are additional costs of M-TEER treatment that we have not included in our analysis such as laboratory costs or maintenance costs. However, the contribution of these factors to the total costs of M-TEER treatment might be low and negligible. The costs of complications were calculated retrospectively, as the rate of complications was low and could not be reasonably addressed by the prospective analysis. However, this cost analysis may be biased by incomplete data due to the retrospective study design. Moreover, it is worth noting that changes in operator experience, device technologies, complication rates, and hospital length of stay may exhibit variations over the extended time span covered by the retrospective analysis. During this period, there was a notable increase in operator experience, and the adoption of modern device technologies has significantly reduced procedure time [25]. However, it is essential to highlight that the rates of complications, which constituted the primary focus of the retrospective analysis, remained consistent over time. This observation aligns with findings from extensive registries that have reported stable complication rates despite the continual growth of M-TEER procedures over the years [26, 27].

We did not evaluate the overall costs (including costs of heart failure hospitalizations in the follow-up) in relation to the overall benefit of M-TEER treatment compared to optimal medical therapy. However, we did conduct a thorough cost analysis for each individual procedure step of the M-TEER procedure hospitalization.

## Conclusion

The major cost driver of M-TEER was the material expenditure, which was mostly triggered by high device costs. The costs of treating patients were similar for the PASCAL and MitraClip systems. M-TEER-related complications are rare but are associated with higher costs, mainly due to prolonged hospitalisation. This analysis provides valuable insights into reducing expenses by modifying the process of M-TEER.

### Supplementary Information

**Additional file 1:** **Supplemental Table 1.** Patient characteristics of MR patients grouped according to degenerative or functional etiology. Values are n (%) or median (interquartile range). * indicates *p* ≤ 0.05 between the groups. **Supplemental Table 2.** Baseline echocardiographic and hemodynamic parameters of MR patients grouped according to degenerative or functional etiology. Values are n (%) or median (interquartile range). * indicates *p* ≤ 0.05 between the groups. **Supplemental Table 3.** Procedural outcome grouped according to degenerative or functional MR etiology. Values are n (%) or median (interquartile range). * indicates *p* ≤ 0.05 between the groups. **Supplemental Table 4.** General costs of M-TEER treatment grouped according to degenerative or functional MR etiology. Values are median (standard deviation). * indicates *p* ≤ 0.05 between the groups. **Supplemental Table 5.** Baseline echocardiographic and hemodynamic parameters of M-TEER patients in retrospective all comer cohort. Values are n (%) or median (interquartile range). * indicates *p* ≤ 0.05 between the groups. **Supplemental Table 6.** Procedural outcome of M-TEER patients in retrospective all comer cohort. Values are n (%) or median (interquartile range). * indicates *p* ≤ 0.05 between the groups.

## Data Availability

The datasets used and analyzed during the current study are available from the corresponding author on reasonable request.
