# Supplementary material for: Comparison of costs associated with transcatheter mitral valve repair: PASCAL vs MitraClip in a real-world setting
Source: BMC Health Serv Res. 2023 Sep 4;23:945. doi: 10.1186/s12913-023-09966-8 (PMC10476289; doi:10.1186/s12913-023-09966-8)
Supplement: Supplementary file 1 — Additional file 1: Supplemental Table 1. Patient characteristics of MR patients grouped according to degenerative or functional etiology. Values are n (%) or median (interquartile range). * indicates p ≤ 0.05 between the groups. Supplemental Table 2. Baseline echocardiographic and hemodynamic parameters of MR patients grouped according to degenerative or functional etiology. Values are n (%) or median (interquartile range). * indicates p ≤ 0.05 between the groups. Supplemental Table 3. Procedural outcome grouped according to degenerative or functional MR etiology. Values are n (%) or median (interquartile range). * indicates p ≤ 0.05 between the groups. Supplemental Table 4. General costs of M-TEER treatment grouped according to degenerative or functional MR etiology. Values are median (standard deviation). * indicates p ≤ 0.05 between the groups. Supplemental Table 5. Baseline echocardiographic and hemodynamic parameters of M-TEER patients in retrospective all comer cohort. Values are n (%) or median (interquartile range). * indicates p ≤ 0.05 between the groups. Supplemental Table 6. Procedural outcome of M-TEER patients in retrospective all comer cohort. Values are n (%) or median (interquartile range). * indicates p ≤ 0.05 between the groups. [file 12913_2023_9966_MOESM1_ESM.docx]

**Supplemental Table 1:** Patient characteristics of MR patients grouped according to degenerative or functional etiology. Values are n (%) or median (interquartile range). * indicates p ≤ 0.05 between the groups

|  | **DMR**  **(n=22)** | **FMR**  **(n=76)** | **p-value** |
| --- | --- | --- | --- |
| **Baseline characteristics**  Age (years)  Female, n (%)  EuroSCORE II (%) | 81.5 (79.3, 85.8)  12 (54.6)  5.1 (3.3, 7.2) | 79.0 (72.0, 83.0)  34 (44.7)  6.5 (4.4, 9.1) | **0.026***  0.472  0.102 |
| STS risk score (%) | 4.5 (3.5, 5.8) | 5.5 (3.6, 7.9) | 0.075 |
| Frailty, n (%) | 11 (50) | 31 (40.8) | 0.442 |
| CCI (points) | 5.0 (4.0, 5.8) | 5.0 (4.0, 7.0) | 0.114 |
| NYHA functional class, n (%)  II  III  IV | 5 (22.7)  14 (63.6)  3 (13.6) | 14 (18.4)  56 (73.7)  6 (7.9) | 0.601 |
| Comorbidities, n (%)  Arterial hypertension  Diabetes mellitus  Coronary artery disease  Previous myocardial infarction  Previous cardiac surgery  ICD/CRT  Atrial fibrillation  Chronic lung disease  Peripheral artery disease  Dialysis for end-stage renal disease  Prior Stroke, n (%) | 19 (86.4)  2 (9.1)  12 (54.5)  1 (4.6)  4 (18.8)  2 (9.1)  17 (77.2)  3 (13.6)  5 (22.7)  0  0 | 62 (81.6)  22 (29)  58 (76.3)  10 (13.2)  27 (35.5)  18 (23.7)  62 (81.6)  16 (21.1)  12 (15.8)  1 (1.3)  6 (7.9) | 1.0  0.089  **0.047***  0.447  0.192  0.228  0.760  0.551  0.524  1.0  0.332 |
| Estimated GFR (ml/min)  NT-proBNP (*1000 pg/ml)  Haemoglobin (g/dl) | 54 (38, 80)  1.11 (0.45, 3.77)  12.0 (11.1, 13.1) | 50 (37, 63)  2.33 (1.19, 4.78)  13.0 (11.7, 13.7) | 0.367  0.082  0.119 |

*MR = mitral regurgitation; DMR = degenerative mitral regurgitation; FMR = functional mitral regurgitation; STS = Society of Thoracic Surgeons; CCI = Charlson Comorbidity Index; NYHA = New York Heart Association; ICD = internal cardiac defibrillator; CRT = cardiac resynchronization therapy; GFR=glomerular filtration rate; NT-proBNP = brain natriuretic peptide*

**Supplemental Table 2:** Baseline echocardiographic and hemodynamic parameters of MR patients grouped according to degenerative or functional etiology. Values are n (%) or median (interquartile range). * indicates p ≤ 0.05 between the groups

|  | **DMR**  **(n=22)** | **FMR**  **(n=76)** | **p-value** |
| --- | --- | --- | --- |
| MR severity, n (%)  Moderate (at rest)  Severe | 1 (4.5)  21 (95.5) | 2 (2.7)  74 (97.3) | 0.538 |
| Left ventricle  Median LVEF (%)  LVEF <40%, n (%)  LVEF 40-50%, n (%)  LVEF > 50%, n (%)  LVEDD (mm)  Left atrium area (cm2)  Transmitral gradient (mmHg)  Vena contracta (mm)  EROA (cm²)  Regurgitation volume (ml) | 60 (52, 62)  1 (4.6)  4 (18.2)  17 (77.2)  52 (46, 55)  23 (18, 33)  2 (2, 3)  7 (6, 8)  0.34 (0.26, 0.65)  52 (43, 103) | 45 (30, 52)  38 (50.0)  16 (21.1)  22 (28.9)  57 (52, 65)  28 (21, 32)  2 (1, 3)  7 (5, 8)  0.29 (0.22, 0.38)  38 (32, 52) | **< 0.001***  **0.019***  0.482  0.328  0.896  0.130  **0.010*** |
| Right Ventricle  TAPSE (mm) | 21 (18, 23) | 17 (15, 19) | **0.014*** |
| Hemodynamic parameters  Cardiac Index  sPAP (mmHg) | 2.3 (1.8, 2.4)  43 (27, 64) | 2.1 (1.8, 2.5)  49 (35, 57) | 0.940  0.737 |

*MR = Mitral regurgitation; DMR = degenerative mitral regurgitation; FMR = functional mitral regurgitation; LVEF = Left ventricular ejection fraction; LVEDD = Left ventricular end-diastolic diameter; RVEDD = right ventricular end-diastolic diameter; EROA = Effective regurgitation orifice area; TAPSE = Tricuspid annular plane systolic excursion; sPAP =* systolic pulmonary artery pressure

**Supplemental Table 3.** Procedural outcome grouped according to degenerative or functional MR etiology. Values are n (%) or median (interquartile range). * indicates p ≤ 0.05 between the groups.

|  | **FMR**  **(n=22)** | **DMR**  **(n=76)** | **p-value** |
| --- | --- | --- | --- |
| Procedure duration (min) | 91 (65, 127) | 94 (80, 127) | 0.377 |
| Conversion to surgery, n (%) | 0 (0) | 0 (0) |  |
| Periprocedural mortality, n (%) | 0 (0) | 0 (0) |  |
| Pericardiocentesis, n (%) | 0 (0) | 0 (0) |  |
| Leaflet device detachment, n (%) | 0 (0) | 0 (0) |  |
| Technical success, n (%) | 22 (100) | 75 (98.7) | 1.0 |
| Minor bleeding complication, n (%) | 1 (4.6) | 2 (2.6) | 0.538 |
| Major vascular complication, n (%) | 0 (0) | 1 (1.3) | 1.0 |
| Myocardial Infarction, n (%) | 0 (0) | 0 (0) |  |
| Pneumonia, n (%) | 1 (4.6) | 2 (2.6) | 0.538 |
| Acute kidney failure, n (%) | 1 (4.6) | 7 (9.2) | 0.679 |
| Stroke 30 days, n (%) | 0 (0) | 0 (0) |  |
| Intrahospital mortality, n (%) | 0 (0) | 0 (0) |  |
| Devices implanted, n (%)  0  1  2  3 | 0 (0)  18 (81.9)  4 (18.1)  0 (0) | 1 (1.3)  48 (63.2)  26 (35.2)  1 (1.3) | 0.408 |
| Degree of MR at discharge, n (%)  mild  moderate  severe  Transmitral gradient at discharge (mmHg) | 16 (72.7)  5 (22.7)  1 (4.6)  4 (3, 5) | 53 (69.7)  22 (28.9)  1 (1.3)  4 (3, 4) | 0.567  0.228 |
| Length of stay in the ICU (d) | 1 (1, 1) | 1 (1, 1) | 0.749 |
| Total length of hospital stay (d) | 6.5 (4, 8) | 5.5 (4, 10) | 0.843 |

*MR = mitral regurgitation; DMR = degenerative mitral regurgitation; FMR = functional mitral regurgitation; ICU =Intensive care unit*

**Supplemental Table 4.** General costs of M-TEER treatment grouped according to degenerative or functional MR etiology. Values are median (standard deviation). * indicates p ≤ 0.05 between the groups.

|  | **DMR**  **(n=22)** | **FMR**  **(n=76)** | **p-value** |
| --- | --- | --- | --- |
| Total costs procedure (€)  Staff costs (€)  Device costs (€)  Costs ICU (€)  Costs general ward (€) | 22142 (22034, 22300)  442 (324, 604)  21000  1469 (1469, 1469)  1880 (1222, 2632) | 22159 (22079, 22279)  453 (381, 602)  21000  1469 (1469, 1469)  1504 (1128, 3384) | 0.549  0.439  1.000  0.749  0.715 |
| Total overall costs (€) | 25721 (24906, 28204) | 25580 (24657, 28234) | 0.432 |

*M-TEER = Transcatheter mitral valve edge-to-edge repair; DMR = degenerative mitral regurgitation; FMR = functional mitral regurgitation; ICU =Intensive care unit*

**Supplemental Table 5:** Baseline echocardiographic and hemodynamic parameters of M-TEER patients in retrospective all comer cohort. Values are n (%) or median (interquartile range). * indicates p ≤ 0.05 between the groups

|  | **Total cohort (n=716)** |
| --- | --- |
| MR etiology, n (%)  Functional MR  Degenerative MR  Mixed disease  Not described | 432 (60.3)  141 (19.7)  52 (7.3)  91 (12.7) |
| MR severity, n (%)  Dynamic moderate to severe  Severe | 149 (20.8)  567 (79.2) |
| Left ventricle  Median LVEF (%)  LVEF <40%, n (%)  LVEF 40-50%, n (%)  LVEF > 50%, n (%)  LVEDD (mm)  Left atrium area (cm2)  Transmitral gradient (mmHg)  Vena contracta (mm)  EROA (cm²)  Regurgitation volume (ml) | 49 (35, 54)  224 (31.3)  140 (19.6)  352 (49.1)  56 (49, 63)  27.8 (23, 32.6)  2.2 (1.9, 3)  7.8 (6.8, 9.7)  0.33 (0.28, 0.44)  48 (38, 61.5) |
| Right Ventricle  RVEDD (mm)  TAPSE (mm) | 43 (38.2, 50.5)  17 (14, 21) |
| Hemodynamic parameters  Cardiac Index  sPAP (mmHg) | 2.1 (1.9, 2.5)  47 (38, 57) |

*M-TEER = Transcatheter mitral valve edge-to-edge repair; MR = Mitral regurgitation; LVEF = Left ventricular ejection fraction; LVEDD = Left ventricular end-diastolic diameter; RVEDD = right ventricular end-diastolic diameter; EROA = Effective regurgitation orifice area; TAPSE = Tricuspid annular plane systolic excursion; sPAP =* systolic pulmonary artery pressure

**Supplemental Table 6.** Procedural outcome of M-TEER patients in retrospective all comer cohort. Values are n (%) or median (interquartile range). * indicates p ≤ 0.05 between the groups.

|  | **Total cohort (n=716)** |
| --- | --- |
| Procedure duration (min) | 92 (72, 119) |
| Technical success, n (%) | 705 (98.5) |
| Devices implanted, n (%)  0  1  2  3  4 | 11 (1.5)  510 (71.2)  185 (25.8)  9 (1.3)  1 (0.1) |
| Transmitral gradient at discharge (mmHg) | 3.4 (2.7, 4.9) |

*M-TEER = Transcatheter mitral valve edge-to-edge repair;*
